# The AI Reviewer: Evaluating AI’s Role in Citation Screening for Streamlined Systematic Reviews

**DOI:** 10.2196/58366

**Published:** 2025-03-28

**Authors:** Jamie Ghossein, Brett N Hryciw, Tim Ramsay, Kwadwo Kyeremanteng

**Affiliations:** 1 Interdepartmental Division of Critical Care Medicine, University of Toronto, Toronto, ON, Canada; 2 Division of Critical Care, Department of Medicine, University of Ottawa, 501 Smyth Road, Ottawa, ON, Canada, 1 (613) 798-5555 ext 16045; 3 Faculty of Medicine, University of Ottawa, Ottawa, ON, Canada; 4 Clinical Epidemiology, Ottawa Hospital Research Institute, Ottawa, ON, Canada; 5 Institute du Savoir Montfort, Montfort Hospital, Ottawa, ON, Canada

**Keywords:** article screening, artificial intelligence, systematic review, AI, large language model, LLM, screening, analysis, reviewer, app, ChatGPT 3.5, chatbot, dataset, data, adoption

## Introduction

Systematic reviews are regarded as one of the highest form of evidence in medical research and are vital for answering clinical questions [1]. However, the conventional systematic review methodology is time-consuming, particularly the manual screening of articles for pertinence [2]. The exponential increase in biomedical literature presents a challenge for researchers to remain updated. Artificial intelligence (AI) has shown promise in various fields [3], with large language models (LLMs) specifically offering capabilities to interpret complex text, which can be leveraged in the systematic review process [4]. We conducted a pilot feasibility study evaluating 5 distinct LLMs in an existing systematic review dataset.

## Methods

### Overview

We compared 5 commonly used LLMs to screen citations from a previously published systematic review on trauma hemorrhage, originally screened by two human reviewers [5]. Of the 1186 total citations, 21 (1.8%) were included for full-text review and 1165 (98.2%) were excluded. We randomly selected 100 excluded citations using Microsoft Excel. Hence, 121 citations (n=21, 17.4% included and n=100, 82.6% excluded) were tested against predefined eligibility criteria using ChatGPT 3.5 (version September 25, 2023), ChatGPT 4 (version September 25, 2023), Google Bard (version 1.15; released on September 2, 2023), Meta Llama 2 (70b parameters, version 2.1.1; released on October 10, 2023), and Claude AI 2 (version 1.3; released on July 11, 2023). We used descriptive statistics to evaluate sensitivity, specificity, and overall accuracy.

### Ethical Considerations

All citations were taken from publicly available, previously published literature. No personal or patient-level data were used, and no identifiers were included. Formal research ethics board approval was therefore not required.

## Results

Among the 121 total citations, the LLMs’ sensitivity (correctly identifying included citations) ranged from 57% to 100%, and specificity (correctly excluding noneligible citations) ranged from 18% to 79%. ChatGPT 3.5 achieved the highest sensitivity (100%) and the highest specificity (79%). Full results are shown in Table 1.

**Table 1.** Performance metrics of large language models in citation screening for systematic reviews, including sensitivity, specificity, and accuracy.

Large language model	Sensitivity, %	Specificity, %	Accuracy, %
ChatGPT 3.5	100	79	83
ChatGPT 4	95	66	72
Google Bard	100	71	77
Meta Llama 2 (70b parameters)	95	18	34
Claude AI 2	57	77	73

## Discussion

In this pilot assessment, selected LLMs demonstrated high sensitivity for identifying relevant studies, with ChatGPT 3.5 and Google Bard reaching 100%. Notably, the specificity varied widely, ranging from as low as 18% for Meta Llama 2 to 79% for ChatGPT 3.5. While some LLMs can be remarkably sensitive for screening articles within our sample, excluding irrelevant citations remains a challenge for certain LLMs. These findings suggest that AI-driven LLMs could be poised to support the screening phase, potentially replacing the second human reviewer and streamlining the often labor-intensive study screening process.

The sample size of 121 citations is a limitation, and findings may not be generalizable to other systematic reviews or inclusion and exclusion criteria. Larger studies, ideally with multiple runs of the same citations, are necessary to capture the probabilistic variability inherent to LLMs. As we only ran each citation through a given LLM once, multiple runs or “prompt engineering” strategies could yield more consistent or refined outcomes when evaluating LLMs. Nonetheless, our study offers a novel approach by directly comparing the performance of multiple LLMs, thus providing insight into how different architectures perform on the same dataset. Future research should explore repeated runs to assess LLM consistency, implement advanced prompt engineering, and investigate the explainability of LLM results.

LLMs have previously been demonstrated to effectively generate Boolean queries for a systematic review literature search [1]. As LLMs evolve further, it is conceivable that they could entirely manage the title and abstract screening. This progress can eventually lead to a fully automated review process, where AI might oversee the search strategy, title and abstract screening, full-text review, data analysis and synthesis, and even drafting and publication. Such automation would epitomize a living systematic review, ensuring evidence is continuously updated as soon as new research is published. As transparency and accountability concerns may arise, a robust ethical framework will be paramount as we navigate the advancements of this technology [6].

## Abbreviations

AI: artificial intelligence
LLM: large language model

## References

[1] Wang S, Scells H, Koopman B, Zuccon G Can ChatGPT write a good boolean query for systematic review literature search?.

[2] Tsafnat G, Glasziou P, Karystianis G, Coiera E (2018). Automated screening of research studies for systematic reviews using study characteristics. Syst Rev.

[3] Hryciw BN, Fortin Z, Ghossein J, Kyeremanteng K (2023). Doctor-patient interactions in the age of AI: navigating innovation and expertise. Front Med (Lausanne).

[4] Kanjee Z, Crowe B, Rodman A (2023). Accuracy of a generative artificial intelligence model in a complex diagnostic challenge. JAMA.

[5] Ghossein J, Fernando SM, Rochwerg B, Inaba K, Lampron J, Tran A (2023). A systematic review and meta-analysis of sample size methodology for traumatic hemorrhage trials. J Trauma Acute Care Surg.

[6] Jha D, Durak G, Sharma V, Keles E, Cicek V, Zhang Z (2025). A conceptual algorithm for applying ethical principles of AI to medical practice. arXiv.

